# Molecular Characterization of Choroideremia-Associated Deletions Reveals an Unexpected Regulation of *CHM* Gene Transcription

**DOI:** 10.3390/genes12081111

**Published:** 2021-07-22

**Authors:** Tiziana Fioretti, Valentina Di Iorio, Barbara Lombardo, Francesca De Falco, Armando Cevenini, Fabio Cattaneo, Francesco Testa, Lucio Pastore, Francesca Simonelli, Gabriella Esposito

**Affiliations:** 1 CEINGE-Biotecnologie Avanzate s.c. a r.l., Via G. Salvatore 486, 80145 Naples, Italy; tiziana.fioretti@gmail.com (T.F.); barbara.lombardo@unina.it (B.L.); defalco@ceinge.unina.it (F.D.F.); armando.cevenini@unina.it (A.C.); lucio.pastore@unina.it (L.P.); 2 Eye Clinic, Multidisciplinary Department of Medical, Surgical and Dental Sciences, University of Campania Luigi Vanvitelli, Via S. Pansini 5, 80131 Naples, Italy; valentina.diiorio@unicampania.it (V.D.I.); francesco.testa@unicampania.it (F.T.); francesca.simonelli@unicampania.it (F.S.); 3 Department of Molecular Medicine and Medical Biotechnologies, University of Naples Federico II, Via S. Pansini 5, 80131 Naples, Italy; fabio.cattaneo@unina.it

**Keywords:** *CHM*, choroideremia, deletion breakpoint, inherited retinal degeneration, REP1, REP2, repeat elements, transcriptional regulation

## Abstract

Choroideremia (CHM) is a X-linked recessive chorioretinal dystrophy due to deficiency of the *CHM* gene product, i.e., Rab escort protein isoform 1 (REP1). To date, gene therapy for CHM has shown variable effectiveness, likely because the underlying pathogenic mechanisms as well as genotype-phenotype correlation are not yet fully known. Small nucleotide variants leading to premature termination codons (PTCs) are a major cause of CHM, but about 20% of patients has *CHM* gene deletions. To improve understanding of the disease mechanisms, we analyzed molecular features of seven deletions involving the *CHM* gene sequence. We mapped the deletion breakpoints by using polymerase chain reaction, sequencing and array comparative genomic hybridization; to identify rearrangement-promoting DNA sequences, we analyzed genomic architecture surrounding the breakpoint regions. Moreover, in some CHM patients with different mutation types, we measured transcript level of *CHM* and of *CHML*, encoding the REP2 isoform. Scattered along the whole *CHM* gene and in close proximity to the deletion breakpoints we found numerous repeat elements that generate a locus-specific rearrangement hot spot. Unexpectedly, patients with non-PTC variants had increased expression of the aberrant *CHM* mRNA; *CHML* expression was higher than normal in a patient lacking *CHM* and its putative regulatory sequences. This latest evidence suggests that mechanisms regulating *CHM* and *CHML* gene expression are worthy of further study, because their full knowledge could be also useful for developing effective therapies for this hitherto untreatable inherited retinal degeneration.

## 1. Introduction

Choroideremia (CHM; MIM #303100) is an X-linked recessive condition characterized by slow progressive degeneration of choroid, photoreceptors and retinal pigmented epithelium. Its prevalence is estimated at 1 in 50,000 males. Affected males develop night blindness in their teenage years, which is followed by loss of peripheral vision and complete blindness at middle age [1]. Very relevant for clinical diagnosis is the evidence that carrier females may exhibit a wide spectrum of disease severity. Indeed, although they are mainly asymptomatic or mildly symptomatic, with funduscopic examination often showing patchy areas of chorioretinal atrophy, a minority of cases presents a severe phenotype, with retinal and choroidal atrophy similar to that observed in affected males [2,3].

CHM is caused by sequence alteration of the *CHM* gene (HGNC:1940; MIM * 300390), which spans a genomic region of about 190 kb on chromosome Xq 21.2 and contains 15 exons [4]. This gene encodes the ubiquitously expressed Rab Escort Protein 1 (REP1) that, similarly to the closely related isoform REP2 (MIM * 118825), is an essential component of the Rab geranyl-geranyl transferase II complex [5,6], which catalyzes prenylation of ras-related Rab GTPases, a group of proteins with key role in of intracellular membrane trafficking regulation [7,8]. Although REP1 deficiency in CHM results in reduced prenylation of a subset of Rabs and affects multiple intracellular trafficking pathways in different cell types, no clinical consequence due to such deficiency has been reported in tissues other than the eye. However, derangement of vesicular trafficking, exocytosis and secretion have been observed in CHM fibroblasts and monocytes, in addition to crystals in peripheral blood lymphocytes and significant abnormalities in plasma fatty acid and red blood cell membrane [9,10].

Currently, prenatal molecular diagnosis is one of the possible prevention options for at risk families because CHM is still an untreatable inherited retinal degeneration [11,12,13,14]. Indeed, although gene therapy has entered late-phase clinical trials, it has shown variable effectiveness, likely because the underlying pathogenic mechanisms as well as genotype–phenotype correlation with respect to onset of symptoms, decline in visual acuity and visual fields are not yet fully known, unlike other retinal dystrophies [13,14,15]. Therefore, any study aimed at elucidating molecular mechanisms underlying CHM could provide useful insight for the development of targeted therapies [16].

More than 420 unique *CHM* pathogenic sequence variants that often abolish REP1 synthesis have been identified in CHM patients (Leiden Open Variant Database—LOVD, https://databases.lovd.nl/shared/genes/CHM; Human gene mutation database, HGMD^®^, accessed on 16 July 2021) [16,17,18,19]. About 20% of patients have chromosomal deletions of variable size that remove single *CHM* exons or eliminate *CHM* and various contiguous genes [19]. No apparent correlation has been found between the size of the deletion and the severity of CHM, however, a few patients with deletions ranging between 5 and 12 Mb had syndromic CHM [20,21,22,23,24].

The relatively high incidence of genomic deletions indicates that *CHM* locus is a hot spot of genomic rearrangements, likely due to the local genomic structure [25]. To date, only a few studies mapped *CHM* deletion breakpoints (BPs) and analyzed local genomic architectural features that might be associated with the generation of this type of copy number variation [4,21]. Therefore, we used a combination of molecular analysis, i.e., polymerase chain reaction (PCR), Sanger’s sequencing and array comparative genomic hybridization (aCGH), to map seven genomic deletions that we identified in unrelated Italian CHM families. We analyzed genomic architecture of the rearranged sequences and, in a few patients, we measured transcript levels of the aberrant *CHM* and of the closely related *CHML* gene (HGNC:1941), encoding the REP2 isoform.

## 2. Materials and Methods

We studied 11 patients (9 males, 2 females) from 7 Italian families affected by CHM with deletions involving *CHM* gene sequences. The diagnosis in males and females was based on clinical and instrumental data [11,26,27]. Diagnostic criteria included a history of night blindness and the typical appearance of the fundus, namely, peripheral pigmentary retinopathy with areas of pigment epithelial and choroidal atrophy [11,26,27]. General anamnesis did not indicate any other significant pathological condition other than CHM. Informed consent was signed by all the patients who approved to undergo the molecular diagnosis, which was performed according to the guidelines for genetic tests approved by the Italian Ministry of Health and by a local Institutional Ethics Committee. Patients also agreed to the use of their clinical and molecular data for scientific research purposes, in anonymous form. Analysis was carried out in accordance with the principles of Declaration of Helsinki.

Genomic DNA was extracted from peripheral blood leukocytes with the Nucleon™ BACC2 kit (GE Healthcare Italia, Milan, Italy). All the 15 *CHM* exons and their flanking regions were amplified by PCR as reported elsewhere [11]. To map intragenic deletion BP, we amplified intronic sequences before and after the deleted exons, according to a previously described strategy [28,29]. Long-range PCR was carried out with the Roche Expand Long Template PCR System (Roche, Mannheim, Germany) to amplify the *CHM* intragenic deletion BPs of family 6 (Fw 5′-CTGATGTCCAGCTGTAGTCTC-3′; Rev 5′-GTAAGTGACAGTCCAGTGTGG-3′) and family 7 (Fw 5′- GGAAGAGGGTAATGAAGTAACG -3′; Rev 5′- CAATGACATCAGAGACAGCC -3′). Fragments spanning the deletion BPs were analyzed by Sanger sequencing and the resulting sequences analyzed by BLAST (http://blast.ncbi.nlm.nih.gov/Blast.cgi, accessed on 21 March 2019) using reference sequences of the Xq21 locus in the human genome assembly 38 (GRCh38/hg38 Human Assembly). To test X-inactivation in carrier females, we carried out methylation-dependent amplification of the polymorphic triplet repeats at the androgen receptor *HUMARA* locus [30].

Total RNA was isolated from peripheral blood leukocytes by using the QIAamp RNA blood mini kit (Italy-QIAGEN S.p.A., Milan, Italy); 100 ng of RNA were reverse transcribed with SuperScript III (Invitrogen, Waltham, MA, USA) and random-hexamers, as previously performed [11]. Real time PCR was carried out in iCycler iQ Real Time PCR Thermal Cycler (Bio-Rad Laboratories S.r.l., Segrate, Italy) with gene-specific (for *CHM* or *CHML*) primers, by using SYBR^TM^ Green Master Mix (Bio-Rad Laboratories S.r.l., Segrate, Italy). Reactions was carried out in triplicate (with duplicate samples for each experiment) in iCycler™ version 3.021 (Bio-Rad Laboratories S.r.l., Segrate, Italy) and the fluorescent signal intensity was recorded and analyzed with the iCycler™ iQ Optical System software v3.0a (Bio-Rad Laboratories S.r.l., Segrate, Italy). Relative gene expression was normalized to *GAPDH* and *ACTB* reference genes and determined using the 2^−ΔΔCt^ method [11,31]. Average values from at least three independent experiments for each sample were graphically reported as fold change and statistical analysis was performed by one-way Student’s *t*-test (for independent samples). To analyze the *CHM* transcript, we used primers REP1^F^: 5′-ATGGCGGATACTCTCCCTTCG-3′ and REP1^R^ 5′-GCTACTATGGAGGAAACTGGG-3′; for *CHML*, primers were REP2^F^ 5′-ATGGCGGACAATCTTCCCACAG-3′ and REP2^R^ 5′-CTATGGAGGAAACTGGGCTAG-3′. Mutation numbering is based on the reference genomic and transcript sequences of *CHM* (NG_009874.2, NM_000390.3).

To perform array comparative genomic hybridization (aCGH) analysis, genomic DNA was analyzed with the Human Genome CGH Microarray kit 4 × 180 K (Agilent Technologies Italia SpA, Milan, Italy). The reference DNA was a pool of genomic DNA from seven normal control samples (Promega, Madison, WI, USA). The aCGH we used contains 170,334 oligonucleotide probes (60-mer), which cover the whole genome with an average spatial resolution of 13 kb. DNA digestion, labeling and hybridization were performed according to the manufacturer’s protocols. Microarrays were scanned on an Agilent G2565CA scanner and image files were quantified using Agilent’s Feature Extraction software [V10.10.1.1]; data were visualized with Agilent’s Genomic WorkBench Standard Edition [V6.5.0.58] (Agilent Technologies Italia SpA, Milan, Italy) [32,33,34].

Bioinformatic analysis of *CHM* genomic sequences were carried out by RepeatMasker [www.repeatmasker.org] and by consulting the Human (Homo sapiens) Genome Browser Gateway of the University of California, Santa Cruz (UCSC) (GRCh38/hg38 Human Assembly; http://genome-euro.ucsc.edu/cgi-bin/hgGateway, accessed on 16 May 2019) [29]. Repeat elements around and within the deleted sequences were aligned by BLAST.

## 3. Results

### 3.1. Molecular Characterization of CHM Deletions

Table 1 lists the deletions identified in the probands (from 7 unrelated families), who belong to a cohort of CHM patients described earlier [11,26].

**Figure 1:**
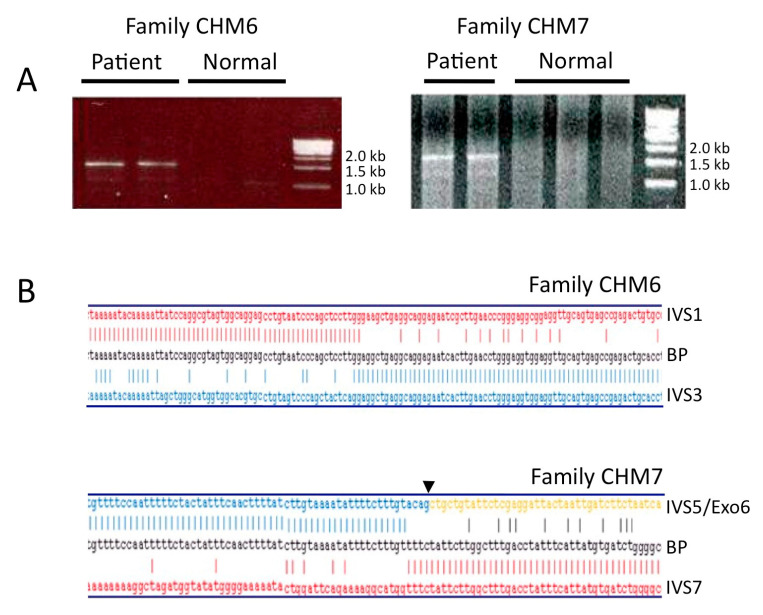
Mapping of intragenic deletion breakpoints in male probands of families CHM6 and CHM7. (**A**) Agarose gel electrophoresis analysis of the *CHM*-specific amplification products obtained by long-range PCR from probands’ genomic DNA and spanning the breakpoint junctions. Unique bands of about 1.5 and 1.7 kb were obtained in the proband of family CHM6 and CHM7, respectively; no amplification was obtained in normal controls. (**B**) Alignment of the residual *CHM* sequences to the reference sequence precisely maps the deletion junction, in CHM6 (top panel) and CHM7 (bottom panel) families. IVS1, intron 1 sequence; IVS3, intron 3 sequence; IVS7, intron 7 sequence; IVS5/Exo6, sequence at the intron 5/exon 6 boundary (arrow); BP, sequence of the deletion breakpoint.

To roughly delimit the extent of the intragenic deletions we found in families CHM6 and CHM7 (Table 1), we amplified *CHM*-specific intronic sequences in the respective affected males and in normal controls. In both cases, we limited the deletion BP within genomic regions no longer than 5 kb and therefore we were then able to amplify, by long-range PCR, the BP junction regions (Figure 1A). Sequence analysis of the resulting amplicons demonstrated that the genomic deletion removing exons 2 and 3 in family CHM6 was 55,962 bp long (Figure 1B, top panel); in family CHM7, a 1635 bp long deletion removed exons 6 and 7 (Figure 1B, bottom panel). In both cases, the deletion caused a shift in the *CHM* transcript reading frame that likely gave rise to a premature termination codon (PTC) [11].

DNA analysis previously performed in family CHM3 revealed the deletion of exon 12; however, leukocytes of affected males lacked the *CHM* transcript [11]. In agreement with the variable spectrum of disease severity observed in CHM carriers [3,27], a 76 year old carrier female affected by a severe phenotype and a 32 year old asymptomatic carrier were also present in this family. The phenotype of the elderly carrier was more severe than that of her affected relatives, so that an incorrect diagnosis of retinitis pigmentosa was initially made. During the familial segregation study, we analyzed RNA extracted from leukocytes of these two females by RT-PCR and sequencing and in both cases we only found the wild type *CHM* transcript. Interestingly, in the elderly carrier, methylation-specific PCR pattern at the *HUMARA* locus was consistent with a random X-inactivation (Supplementary Figure S1), thereby suggesting that her severe phenotype was not due to a somatic unbalanced lyonization favoring expression of the X chromosome that carried the mutated *CHM* gene. This result is in line with previous data that do not support a correlation between X chromosome inactivation status and abnormal retinal phenotype in CHM carrier females [35].

We previously reported that, in family CHM4, the genomic deletion removing *CHM* exons 10–15 produced an aberrant transcript that retained exons 1–9 and part of intron 9, presumably leading to a truncated REP1 (p.Cys416Ter) [11,26]. In family CHM5, the genomic deletion removed *CHM* exons 1–12 and extended upstream the putative *CHM* promoter region [36]; accordingly, RT-PCR did not detect any specific transcript in leukocytes of the male proband of CHM5 family.

**Figure 2:**
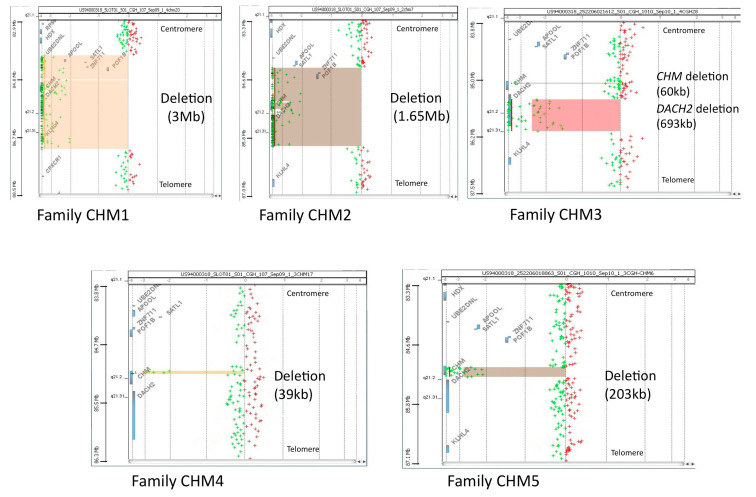
Array CGH profile of chromosome Xq21.3 in index cases. Scattered plot analysis mapped the extent of the deletion in probands of families CHM1 (~3 Mb), CHM2 (~1.65 Mb), CHM4 (~39 kb) and CHM5 (~203 kb); in family CHM3, aCGH confirmed the deletion that removed exon 12 of *CHM* and identified an additional wide deletion of ~693 kb that involved most sequence of the adjacent *DACH2* gene. Each cross represents a single probe (horizontal shift to left of 0 indicates a deleted sequence). Log2 (ratio) was plotted for all the oligonucleotide probes, based on their chromosome positions. Aberration calls identified by ADM-2 algorithm are shown as shaded areas.

To define the extent of the widest deletions identified in our families, we performed aCGH analysis (Figure 2), which is able to successfully detect in a single experiment copy number changes across the whole genome [32,33,34]. In family CHM1, array limited the deletion to about 3 Mb that removed *UBE2DN* (pseudogene), *APOOL, SATL1, ZNF711, POF1B, CHM, DACH2* and *KLHL4* genes. In family CHM2, the deletion was about 1.65 Mb long and encompassed *SATL1, ZNF711, POF1B, CHM* and *DACH2*. In family CHM4, the deletion that removed exons 10–15 was about 34 kb long, started in IVS9 and ended in the 3′UTR of *CHM*, just before the polyadenylation signal. In family CHM5, aCGH showed that the deletion removed about 153 kb of *CHM*, including exons 1–12 and about 33 kb of its 5′ flanking sequences. In family CHM3, aCGH confirmed the deletion of *CHM* exon 12 and surprisingly revealed an additional structural variation, i.e., a deletion that started in intron 1 and removed all the downstream sequences of *DACH2*, a *CHM* contiguous gene.

### 3.2. Analysis of the Genomic Architecture Flanking the BP Junctions

Once the approximate genomic positions of our BPs were determined, we analyzed junction-sequence signatures to explore why the *CHM* locus and the surrounding genomic regions are so prone to rearrangements. First, we performed in silico analysis of the Xq21.2 locus to search for repeated sequences potentially leading to genomic instability. RepeatMasker and UCSC genome browser-based analysis of the largest deleted region (chrX:84,831,307–87,941,186) in our patients revealed 4473 interspersed repeats, mainly long interspersed elements (i.e., LINE1, LINE2), short interspersed elements (i.e., Alu, MIR), long terminal repeats, single tandem repeats. Along the X chromosome, the *CHM* gene (chrX: 85,861,180–86,047,562; NC_000023.11) has inverted orientation (minus strand).

Numerous and different types of repeated elements are also scattered along the whole *CHM* gene and in close proximity to the breakpoints of the deletions detected in our families (Supplementary Figure S2).

Table 2 summarizes high identity interspersed repeats that lie within the genomic regions proximal and distal to the deletion BPs of our CHM patients and their relative percentage of sequence identity, as determined by RepeatMasker and BLAST analysis, respectively. Moreover, we used BLAST to look for sequence identity in the regions surrounding the BPs of each deletion. Notably, the BP junction regions of the five largest deletions contained sequence stretches of various extents (from 13–80 to 200–500 bp long) that share from 65% to 100% identities (Table 2, last column).

Lastly, we compared by BLAST the whole sequences of *CHM* (NC_000023.11; chrX:85861180–86047562) and *DACH2* (NC_000023.11; chrX:86148451–86832602) and found that the two genes contain at least 12 large sequence stretches (ranging from ~2000 to ~5000 bp) that share from 77% to 92% of identities.

### 3.3. Analysis of CHM and CHML Transcript Level

**Figure 3:**
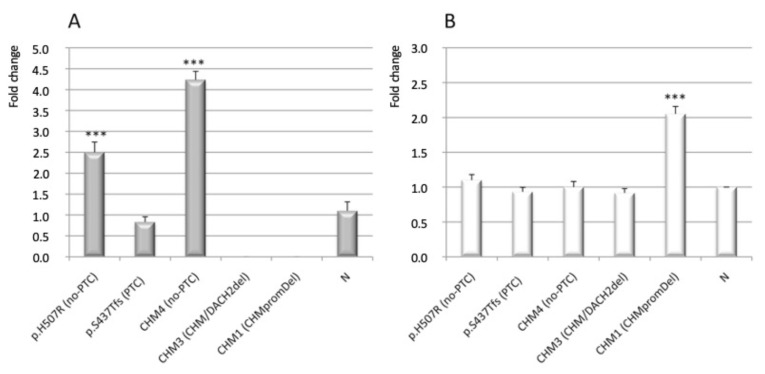
Quantitative RT-PCR analysis of *CHM* and *CHML* transcript levels in CHM patients with specific pathogenic variants (see text). (**A**) Transcript level of *CHM* is significantly higher in the patient with the missense variant p.His570Arg and in the CHM4 patient, both expressing no-PTC mRNAs, than in normal controls (N); patient with a classic PTC-associated pathogenic variant (p.Ser437Trpfs) has *CHM* mRNA level similar to normal controls; *CHM* expression was null in patients CHM3 (*CHM*/*DACH2* del) and CHM1 (a 3 Mb deletion that removed the whole *CHM* gene including its promoter). (**B**) In patient CHM1, who lacks the *CHM* gene promoter and in the other CHM patients tested, transcript level of *CHML* is twice and equal to the normal controls, respectively. Error bars represent the means of three independent experiments; statistical significance was calculated by one-way two-tailed *t*-test for independent samples (*** *p* < 0.005).

To further study molecular consequences of the mapped deletions, in the three male patients for whom RNA was available (namely CHM1, CHM3, CHM4), we analyzed residual *CHM* transcript level. Results obtained from the deleted patients were compared to those obtained from selected reference males, i.e., a patient with a typical PTC frameshift variant (p.Ser437Thrfs), one with a no-PTC variant, namely the missense p.His507Arg, and three normal males. Of note, also the deletion sparing the *CHM* polyadenilation site (p.Cys416Ter), which was detected in the CHM4 proband, is a no-PTC variant. We confirmed absence of *CHM* expression in the male patient of family CHM3 (with the *CHM/DACH2* complex deletion), whereas mRNA level of the patient with the PTC variant p.Ser437Thrfs was comparable to the normal (Figure 3A). Surprisingly, in leukocytes of both patients having the no-PTC variants p.His507Arg and p.Cys416Ter, the respective aberrant *CHM* transcript levels were 2.5 and 4.0 fold higher than both normal and PTC mRNAs (Figure 3A).

As literature reports that the phenotypic variation in CHM may in part be explained by the degree to which the absence of REP1 can be compensated by other prenylation proteins such as REP2 [14], we also analyzed expression of *CHML*, encoding the REP2 isoform, in the same patient group. Notably, *CHML* mRNA expression was comparable to normal in the CHM3 and CHM4 probands and in the affected and unaffected reference subjects (see above), but was unexpectedly higher (2.0 fold) than normal in the analyzed proband of family CHM1, who had a deletion removing the whole *CHM* gene and its flanking regions (Figure 3B).

## 4. Discussion

Pathogenic variants in the *CHM* gene result in complete loss of REP1, which is a typical feature of CHM cells [10,16,17,18,19]. Genomic deletions involving *CHM* are loss-of-function defects that have been detected in about 15–20% of CHM patients [19,20,21,22,23]. Here, we report molecular characterization of seven large deletions that remove in part or all the *CHM* gene in Italian patients with choroideremia (Table 1). Similar deletions are listed in the *CHM*-specific LOVD database, which in the July 2021 update reports 84 different ones: 28% of them remove the whole gene and its flanking regions, 30% has only one BP that falls within the *CHM* gene, the remaining are deletions with two intragenic BPs.

Genomic sequences that include tandem repeats and interspersed repeats are prone to deletion and duplication events [25]. Interestingly, bioinformatic analysis revealed an abundance of repeated elements scattered along the *CHM* gene locus (Supplementary Figure S2, bottom panel); in addition, local sequence identities are present in proximity of the BP regions of the deletions identified in our families (Table 2). In particular, three BP regions contain Alu elements, three contain LINEs, and one has LTRs; numerous STRs are present, in at least five cases (Table 2).

Notably, intragenic BPs of the deletions reported in LOVD are scattered throughout the *CHM* gene, without evidence of BP hot spots (Supplementary Figure S2, top panel) and, accordingly, recurrent deletions have never been reported in CHM patients, including ours. These observations suggest that a non-homologous end joining mechanism mediated by the spread repetitive elements can be responsible for the deletion susceptibility of the whole *CHM* gene locus [25]. As a non-homologous end joining mechanism may also cause sequence duplication, this type of rearrangement should be taken into account during the molecular diagnosis of CHM [20]. Therefore, multiple ligation-dependent probe amplification or/and aCGH analysis is suggested for CHM patients without SNVs or large deletions [18,37]. Obviously, any data obtained from mRNA analysis may further warrant such in-depth diagnostic study.

In agreement with data reported in the *CHM*-specific LOVD database, two of our seven families (28%) have deletions that involve the whole *CHM* and some contiguous genes (Figure 2). In particular, the nullisomic regions deleted in families CHM1 and CHM2 contain genes that exert different functions, such as the *ZNF711* gene, which encodes a zinc-finger sequence-specific DNA binding factor [38,39,40]. Additionally deleted in both our families are *POF1B,* which seems to play a role in the etiology of premature ovarian failure [41], and *DACH2,* with an important role in the regulation of brain and limb development and presumed to be implicated in syndromic mental retardation [41,42].

A few earlier cases of CHM associated with extra-ocular symptoms were attributed to contiguous gene deletions at Xq21 locus [23,24,43]. Interestingly, a large deletion involving *CHM* and the neighboring *POU3F4* and *ZNF711* genes has been previously identified in a CHM patient with hearing impairment and developmental disability [38]. In agreement, two families with nonspecific mental retardation and truncating alterations in the *ZNF711* gene were also reported [39,40]. As our male patients with contiguous gene deletions seemed to be affected by isolate choroideremia [11,26], we conclude that the genes located within the deleted genomic regions might have only indirect or subtle effects on the patients’ extra-ocular phenotype [43]; consequently, we can reasonably state that loss of *ZNF711* is not associated with mental retardation, in our patients.

Small nucleotide changes leading to nonsense or frameshift variants are frequent loss-of-function defects in *CHM*. However, the nonsense-mediated mRNA decay (NMD) pathway, which usually detects and degrades mRNAs carrying mutations that lead to PTCs, does not appear to be an efficient surveillance mechanism in the leukocytes of CHM patients [11,44,45]. As consequence, the main loss-of-function effect of *CHM* nonsense or frameshift variants and of partial intragenic deletions is not absence of the aberrant mRNA, but rather the production of truncated proteins that are prone to cell protein degradation [27,45]. In line with this observation, CHM patients usually express detectable level of the aberrant *CHM* transcript [11,18,45]. Therefore, unusual was the case of the complex deletion that removes the *CHM* exon 12 and most sequences of the adjacent *DACH2* gene, in our family CHM3, because in leukocytes of the analyzed probands we did not find the expected aberrant *CHM* mRNA lacking exon 12. We consider it unlikely that NMD completely degrades such putative deleted transcript, as an mRNA lacking exon 12 was previously isolated in another CHM patient [46]. As *CHM* and *DACH2* are adjacent head-to-head tandem arranged genes (Supplementary Figure S3) with the respective transcription start sites about 100 kb apart, we speculate that the deletion of *DACH2* could play a role in *CHM* expression. Indeed, it is conceivable that *cis*-acting elements within the *DACH2* sequences deleted in this family may be involved in transcriptional regulation of *CHM*, as occurs for other genes [47]. Further studies are required to identify potential, to date unknown, long-range *cis*-regulatory elements of *CHM*, whereas a proximal *cis*-regulatory element has been rather recently identified [36]. On this basis, we do not exclude that rare CHM patients who test negative to *CHM* mutation might have deletion of *DACH2*.

In this study, we also analyzed the expression of the *CHM* and *CHML* transcripts in patients who had different types of pathogenic *CHM* sequence variants. Due to the rarity of the disease, the uniqueness of patients with specific genotypes and the difficulty to collect fresh blood sample from low vision or blind patients often living far from our diagnostic centers, this analysis is limited to a small number of patients. Nevertheless, our data surprisingly associated two very rare no-PTC sequence changes (i.e., p.His507Arg and p.Cys416Ter), a type of variants not subjected to NMD, with increased *CHM* transcription, which could be ascribed to the activation of an auto-regulatory loop generated to overcome, although unsuccessfully, REP1 deficiency in CHM leukocytes. As consequence, in CHM patients with PTC-associated variants, residual expression of aberrant *CHM* transcripts may depend on a balance between forced/increased gene transcription and NMD, which apparently makes the latter poorly effective. Additionally, the finding that affected males of family CHM1, who have a deletion that removes *CHM* and, most importantly, its promoter, showed significant increased expression of *CHML* is interesting. Based on this isolate observation and on previous evidence that knockdown of *CHML* upregulates the expression of *CHM* in HeLa cells [48], we suggest that transcription of *CHM* and *CHML* may respond to common regulatory mechanisms. Interestingly, our previous clinical investigation revealed that probands of CHM1 family had a more severe loss of visual function at younger age than the other CHM patients evaluated [26]. Therefore, we speculate that *CHML* overexpression might even have an adverse impact on disease progression. This observation could be relevant during selection of subjects to be enrolled in clinical trials, as well as in the follow-up of experimental therapy on disease progression [49,50].

## 5. Conclusions

Enhanced rearrangement vulnerability of the *CHM* gene locus can be attributed to the abundance of repetitive elements that, by mediating non-homologous end joining mechanisms, trigger gene deletions, which indeed represent about 28% of the disease-causing gene variants found in CHM patients. Moreover, our data shed some light on the possible mechanisms that regulate *CHM* gene transcription and ultimately provides hints that transcription of *CHM* and *CHML* could respond to common regulatory mechanisms. Obviously, further study is needed to support our conclusion.

Overall, our study provides new insight into the molecular basis of CHM, which can be also relevant for diagnosis and treatment of patients affected by this degenerative eye disorder.

## Figures and Tables

**Table 1:** *CHM* deletions identified in Italian patients with choroideremia.

Family/Proband	Deleted Exon	Genomic Deletion	Protein ^a^
CHM1	1–15	chrX:g.(84,831,307_84,886,530)_(87,891,525_87,941,186)del	p.0
CHM2	1–15	chrX:g.(85,123,800_85,156,157)_(86,816,070_86,832,590)del	p.0
CHM3	12	**chrX: g.[(85,879,855_85,894,160)_(85,894,219_85,901,695)del; (86,233,417_86,253,764)_(86,946,402–87,002,613)del] ^b^**	p.0
CHM4	10–15	chrX:g.(85,862,591_85,864,525)_(85,903,612_85,909,335)del	p.Cys416Ter
CHM5	1–12	chrX:g.(85,890,383_85,894,156)_(86,097,388_86,111,691)del	p.0
CHM6	2–3	chrX:g.85,981,356_86,037,319del	p.Leu18Lysfs*10
CHM7	6–7	chrX:g.85,957,346_85,958,981del	p.Leu234Aspfs*4

Deletion numbering is reported to the GRCh38/hg38 Human Assembly; NC_000023.11; ^a^ NP_0000381.1; ^b^ *CHM* plus *DACH2* deletion. In bold, the new deletion characterized in this study.

**Table 2:** High identity repeated elements surrounding the choroideremia-associated deletion breakpoint regions within the *CHM* locus.

Family/Proband	Deletion Extent	Lost Exons	Proximal RE	Distal RE	RE Class	Sequence Identity (%)
CHM1	~3 Mb	1–15	L1PA10, L1PA12	2L1PA14, STR	LINE, STR	78
CHM2	~1.65 Mb	1–15	AluY, AluJb, STR	AluSx	SINE, STR	70–80
CHM3	~60 kb	12	L1MA9	L1PA16	LINE	69
	~670 kb [*DACH2*]	2–12 *	L1PA15, STR	L1PA2, STR	LINE, STR	76
CHM4	~34 kb	10–15	AluSx	AluSx1	SINE	81
CHM5	~235 kb	1–12	THE1D-int	THE1A-int, STR	LTR, STR	78
CHM6	~55 kb	2–3	AluSq	AluSx	SINE	84
CHM7	~1.6 kb	6–7	MER46C	MER3, MIRc	SINE, STR	None

LINE, long interspersed element; SINE, short interspersed element; LTR, long terminal repeat; RE, repeated element; STR, single tandem repeat. * *DACH2* transcript variant 1 [GenBank # NM_053281.3].

## Supplementary Materials

The following are available online at https://www.mdpi.com/article/10.3390/genes12081111/s1, Figure S1: X-chromosome inactivation patterns at the *HUMARA* locus in the affected elderly female of family CHM3, Figure S2: Position of deletion breakpoints and types of repeated elements scattered along the *CHM* gene, Figure S3: Organization of *CHM* and adjacent genes on chromosome Xq21.3 (NC_000023.11).
