# Supplementary material for: Molecular Characterization of Choroideremia-Associated Deletions Reveals an Unexpected Regulation of CHM Gene Transcription
Source: Genes (Basel). 2021 Jul 22;12(8):1111. doi: 10.3390/genes12081111 (PMC8392058; doi:10.3390/genes12081111)
Supplement: Supplementary file 1 [file genes-12-01111-s001.zip › genes-1280905-supplementary.pdf]

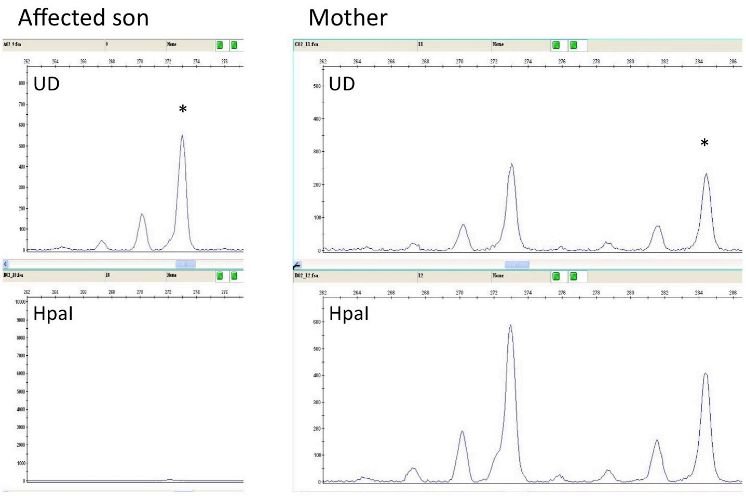

Figure S1. X-chromosome inactivation patterns at the *HUMARA* locus in the affected elderly female of family CHM3. In the affected son, amplification of *Hpa*II-undigested (UD) DNA revealed one allele from his X chromosome (*); this unmethylated allele was not amplified after *Hpa*II digestion of DNA. In the mother, peak intensity quantification of the two maternal alleles detected before (UD) and after DNA digestion with *Hpa*II indicates a random X-chromosome inactivation (60% vs 40%).

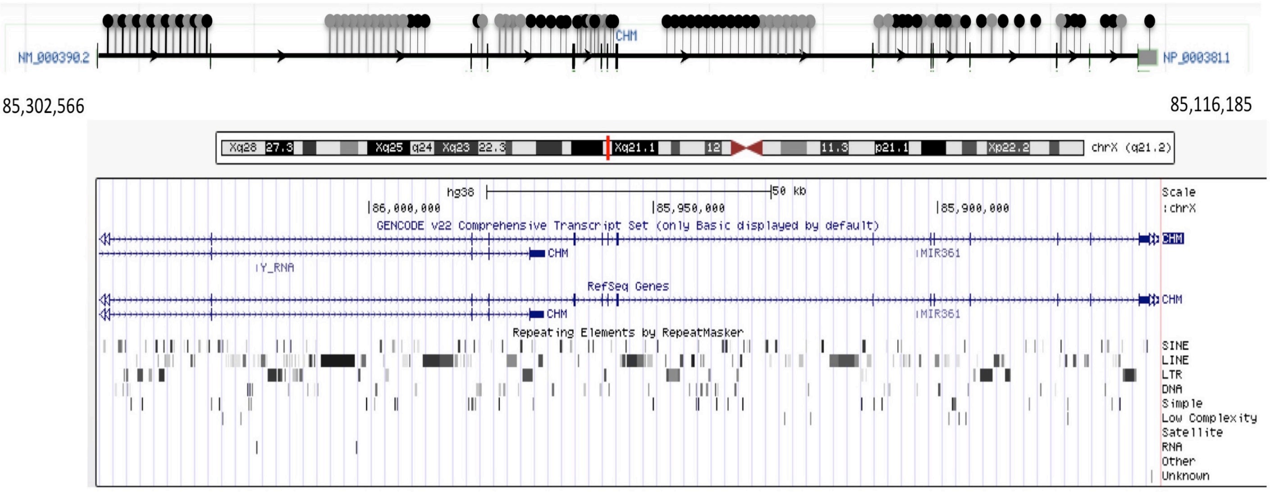

Figure S2. Position of deletion breakpoints and types of repeated elements scattered along the *CHM* gene. Top panel roughly indicates start (grey sticks) and end (black sticks) positions of the large deletions with at least one intragenic breakpoint, as currently reported in the LOVD database (June 2021 update). Bottom panel is a screenshot of the UCSC genome browser-based RepeatMasker analysis showing the different types and position of repeated elements located within the *CHM* gene.

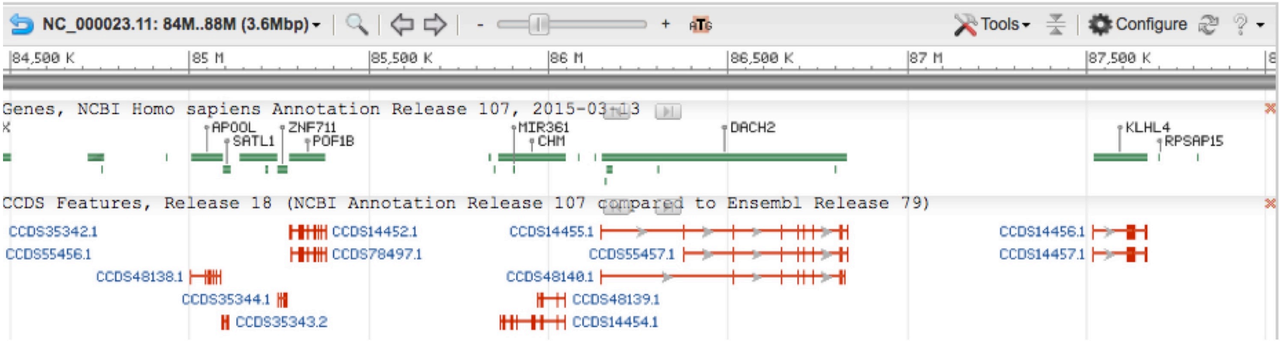

Figure S3. Organization of *CHM* and adjacent genes on chromosome Xq21.3 (GenBank # NC_000023.11). Relative positions of *CHM* and contiguous genes that are involved in the deletions we in our CHM patients (http://www.ncbi.nlm.nih.gov/gene/1121). For each gene, all the known isoforms with the corresponding Ensembl (http://www.ensembl.org/index.html) codes (CCDS) are shown. *CHM* and *DACH2* are adjacent, head-to-head tandem arranged genes.
